# Molecular characterization of the apical organ of the anthozoan *Nematostella vectensis*

**DOI:** 10.1016/j.ydbio.2014.11.019

**Published:** 2015-02-01

**Authors:** Chiara Sinigaglia, Henriette Busengdal, Avi Lerner, Paola Oliveri, Fabian Rentzsch

**Affiliations:** a Sars Centre for Marine Molecular Biology, University of Bergen, Thormøhlensgt 55, 5008 Bergen, Norway; b Department of Genetics, Evolution and Environment, University College London, Gower Street, London WC1E 6BT, UK

**Keywords:** Apical organ, *Nematostella*, Sea urchin, Cilia, Life cycle, FGF

## Abstract

Apical organs are sensory structures present in many marine invertebrate larvae where they are considered to be involved in their settlement, metamorphosis and locomotion. In bilaterians they are characterised by a tuft of long cilia and receptor cells and they are associated with groups of neurons, but their relatively low morphological complexity and dispersed phylogenetic distribution have left their evolutionary relationship unresolved. Moreover, since apical organs are not present in the standard model organisms, their development and function are not well understood. To provide a foundation for a better understanding of this structure we have characterised the molecular composition of the apical organ of the sea anemone *Nematostella vectensis.* In a microarray-based comparison of the gene expression profiles of planulae with either a wildtype or an experimentally expanded apical organ, we identified 78 evolutionarily conserved genes, which are predominantly or specifically expressed in the apical organ of *Nematostella*. This gene set comprises signalling molecules, transcription factors, structural and metabolic genes. The majority of these genes, including several conserved, but previously uncharacterized ones, are potentially involved in different aspects of the development or function of the long cilia of the apical organ. To demonstrate the utility of this gene set for comparative analyses, we further analysed the expression of a subset of previously uncharacterized putative orthologs in sea urchin larvae and detected expression for twelve out of eighteen of them in the apical domain. Our study provides a molecular characterization of the apical organ of *Nematostella* and represents an informative tool for future studies addressing the development, function and evolutionary history of apical organ cells.

## Highlights

• Microarray-based characterization of the apical organ in *Nematostella vectensis*.
• Apical organ expression of 78 conserved genes confirmed by in situ hybridization.
• Putative orthologous genes in sea urchin are also expressed in its apical organ.
• The data present a molecular signature for comparative studies of apical organs.

## Introduction

**Fig. 1:**
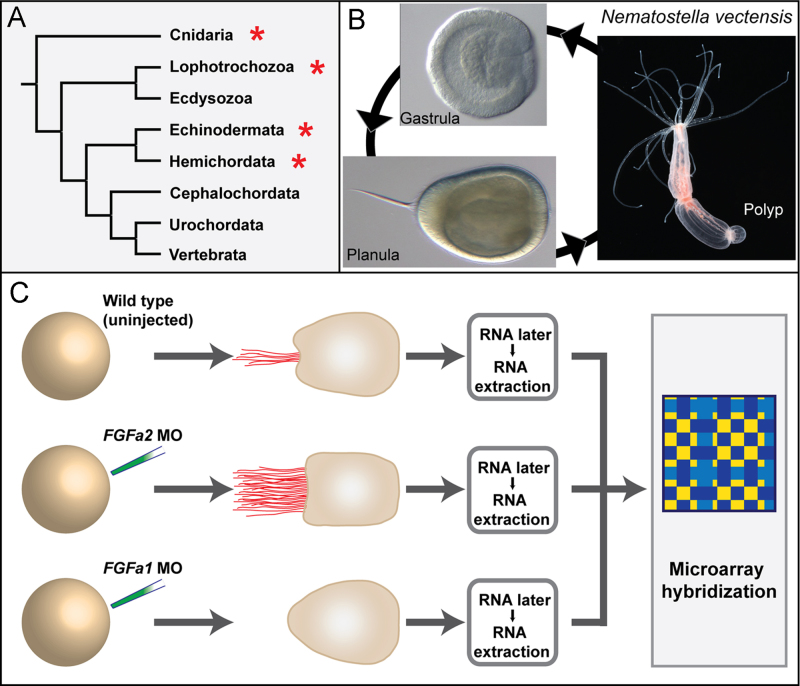
*Nematostella vectensis* as a model for the identification of apical organ genes (A) Apical organs are found in the developing stages of diverse invertebrates: Cnidaria, Lophotrochozoa, Echinodermata and Hemichordata (red asterisks). (B) *N. vectensis* is a representative of the Anthozoa, the sister group to all other cnidarians. Anthozoa is the only class of Cnidaria where apical organs with long cilia have been described. Embryonic development comprehends a swimming stage, the planula larva, which bears a tuft of long cilia at the aboral pole. The apical organ disappears after about one week of development, when the larva settles and develops the tentacles. (C) Experimental design for the microarray analysis. Apical organs (red tuft in the drawing) were manipulated by injecting antisense morpholinos directed against two FGF ligands with opposite functions: *NvFGFa1* MO produces larvae lacking an apical organ, while *NvFGFa2* MO leads to larvae with an expanded organ. The samples were preserved until total RNA was extracted from the different conditions (including the control wild type). The transcription profiles of the three phenotypes were compared in a microarray analysis.

Apical organs are found in the larval stages of phylogenetically diverse animal groups (Fig. 1A) such as anthozoan cnidarians, protostomes (e.g. molluscs and annelids) and deuterostomes (e.g. echinoderms and hemichordates). The functions of apical organs are not well understood and may vary between taxa. Given their temporal restriction to larval stages, apical organs have been proposed to play an important role in the detection of settlement cues and for the induction of metamorphosis, presumably acting as chemosensory and/or mechanosensory structures. Such a function has been confirmed by functional approaches in only few species (Conzelmann et al., 2013; Hadfield et al., 2000; Kempf et al., 1997; Rentzsch et al., 2008; Voronezhskaya and Khabarova, 2003; Voronezhskaya et al., 2004). The regulation of this life cycle transition not only represents a key developmental, but also an important ecological role, since the recruitment to the substrate and the metamorphosis of swimming larvae contribute to the shaping of benthic communities (e.g. Shikuma et al., 2014; Siboni et al., 2014; Witt et al., 2011). Moreover, apical organs are thought to regulate the activity of ciliary bands and musculature in some bilaterians and thus likely contribute to their locomotion (Croll and Dickinson, 2004; Goldberg et al., 1994; Satterlie and Cameron, 1985).

Apical organs are often considered the only larvae-specific organ, and according to this assumption, the question whether they share a common evolutionary origin has important implications for the evolution of metazoan life cycles. It has been proposed that apical organs could represent the simple brain of a gastrula-like ancestor of cnidarians and bilaterians (see Nielsen, 2005a). If this holds true, this common ancestor would have developed through a larva-like stage and adult stages were either added multiple times independently or have diversified drastically during evolution. If instead apical organs are not homologous, this would lend support to the hypothesis that the development of the common ancestor did not comprise a larva-like stage and that larvae in different taxa are the product of convergent evolution (Raff, 2008; Sly et al., 2003). However, the distinction of direct vs indirect development (i.e. with or without larval stage) can be difficult and, accordingly, using potential homology of apical organs to infer the evolution of life cycles is not straightforward.

Two distinct aspects can be considered when discussing the potential homology of apical organs. The first one is their position: apical organs are generally located at the pole opposite to the gastrulation site (Nielsen, 2012), in a specific territory of the embryo called the apical domain (Lacalli, 1994; Nielsen, 2012). This position tightly couples the development of apical organs to the patterning of the apical-blastoporal axis.

Besides this shared position, diagnostic morphological characters of bilaterian apical organs are rather limited: in general, a tuft of long cilia emerging from mono- or multiciliated cells is present, accompanied by flask-shaped receptor cells, often positive for serotonin or FMRFamide immunoreactivity and connected to the larval nervous system by a plexus of neurites (Byrne et al., 2007; Hay-Schmidt, 2000; Nakajima et al., 1993; Richter et al., 2010). The morphology, number and arrangement of nerve cells associated with apical organs can vary considerably (e.g. Altenburger and Wanninger, 2010; Dickinson and Croll, 2003; Hay-Schmidt, 2000; Kempf et al., 1997; Temereva and Wanninger, 2012; Voronezhskaya et al., 2002). In deuterostomes, the cell bodies of the apical organ-associated neurons are typically located in or close to the apical portion of the ciliary bands and can be organized into bilaterally arranged ganglia (Byrne et al., 2007; Nielsen, 2012). Generally, larvae swim with their apical organs pointing forward, prior to settling on a substrate and undergoing metamorphosis—at which point the apical organ degenerates (Gifondorwa and Leise, 2006; Hinman et al., 2003; Nielsen, 2005b).

Since Cnidaria are the sister group to Bilateria, their apical organs are particularly relevant for evolutionary comparisons (Hejnol et al., 2009; Philippe et al., 2009). Based on similarities in the genetic control of the development of the bilaterian anterior/apical pole and the aboral pole of *Nematostella vectensis* homology of these two domains has recently been proposed (Marlow et al., 2014; Santagata et al., 2012; Sinigaglia et al., 2013), suggesting that apical organs in a cnidarian and in bilaterians develop from homologous territories. However, these studies addressed the homology of the apical organ itself only to a very limited extent. Cnidarian apical organs with a ciliary tuft have only been identified in anthozoan, and not in medusozoan planulae. They have been described as a set of columnar cells with basal nuclei, with a cytoplasm filled with various undetermined vesicles, and an underlying plexus of neurites (Chia and Koss, 1979; Nakanishi et al., 2012) but, in contrast to bilaterians, distinct nerve cells associated with the apical organ have not been identified. In addition to the long cilia-bearing cells, the apical organ of *N. vectensis* contains two types of gland or secretory cells (Nakanishi et al., 2012). Clearly, the paucity of comparable morphological characters both in bilaterians and cnidarians complicates inferences about the homology of apical organs. Moreover, possible homologies may not be found at the level of the whole apical organ, but rather at the level of individual cell types (Arendt, 2008; Marlow et al., 2014)

Accordingly, a molecular characterization of the cells constituting the apical organ is necessary in order to improve our understanding of this structure from both a functional and evolutionary perspective. Ideally, such a molecular characterization would include genes which control the development of the apical organ cells (i.e. signalling molecules and transcription factors), and genes that define specific cellular structures and processes (e.g. cytoskeletal components or metabolic genes). Developmental regulators are frequently used to test potential homology of characters (e.g. body parts or cell types) in different organisms with the assumption that a common evolutionary origin (i.e. homology) should be reflected in a shared developmental program (Davidson and Erwin, 2006; Wagner, 2007). However, substantial parts of a particular developmental program can be re-employed for the formation of evolutionarily unrelated characters. This is most evident for signal transduction pathways which often control the development of a plethora of characters in a spatially and temporally distinct manner (e.g. Hikasa and Sokol, 2013; Koles and Budnik, 2012; Lim and Nusse, 2013; Regard et al., 2012; von Maltzahn et al., 2012). Thus, information about the differentiated structure is an important aspect of a homology assumption. This information is typically derived from morphological analyses, but can also be provided by a molecular characterization, e.g. by the structural or physiological components that are present in the structure. For the comparison of cnidarian and bilaterian apical organs, information about their development and about the apical organ cells is currently scarce, mainly due to the absence of apical organs in the classical model organisms for molecular developmental biology, such as *Drosophila*, *Caenorhabditis elegans* or vertebrates. Within bilaterians, a set of conserved transcription factors and signalling molecules has been identified that is expressed in the apical organ regions in the trochophore larvae of the annelid *Platynereis dumerilii*, in sea urchin embryos and in the hemichordate *Saccoglossus kowalewskii*. These similarities have been used to propose the homology of bilaterian apical organ cell types and their development (Marlow et al., 2014).

In the present study we aim to provide a molecular fingerprint of the apical organ in the anthozoan *N. vectensis* (Fig. 1B)*,* using an approach which is not based on candidate genes. The starting point of this study is the involvement of FGF signalling in the formation of the apical organ, and in particular of two FGF ligands that are co-expressed at the aboral pole and which have opposite functions: *NvFGFa1* promotes the formation of the apical organ and the associated tuft of cilia, while *NvFGFa2* restricts the size of the apical organ, as demonstrated by gene knockdown experiments (Rentzsch et al., 2008). Importantly, *NvFGFa1* and *NvFGFa2* function specifically in the formation of the apical organ, but not in the definition of the aboral territory (Sinigaglia et al., 2013). The phenotypes resulting from the injection of morpholino (MO) antisense oligonucleotides directed against the two FGF ligands are easily discernible at larval stage: *NvFGFa1* MO produces embryos without a ciliary tuft, while *NvFGFa2* MO produces embryos with a strongly expanded tuft (Rentzsch et al., 2008). This observation provided us with a unique tool to identify genes that are expressed in the apical organ by comparing the gene expression profiles of these two situations by microarray analysis (see Fig. 1C). Since previously identified transcription factors and signalling molecules with potential roles in the development and/or the differentiation of the apical organ remain expressed throughout planula stage (e.g. Magie et al., 2005; Matus et al., 2007; Pang et al., 2004; Sinigaglia et al., 2013) the expression analysis at this relatively late stage should in principle allow the identification of both developmental regulators and structural genes.

The 78 genes recovered with this approach provide a basis for a detailed characterization of the cells constituting the apical organ of *Nematostella*. Moreover, by analysing the expression of a subset of these identified apical organ genes in sea urchin, we demonstrate the utility of this dataset for comparative studies.

## Materials and methods

### *Nematostella* culture

*N. vectensis* was maintained and induced to spawn in the Sars Centre facility, as described previously (Fritzenwanker and Technau, 2002; Hand and Uhlinger, 1992).

### Design and analysis of microarray

The experimental design is illustrated in Fig. 1C. *Nematostella* eggs were injected after fertilization, with either morpholino for *NvFGFa1* or for *NvFGFa2*, as in Rentzsch et al. (2008). Planulae with the expected phenotype and without general developmental abnormalities were selected after 72 h of development at room temperature, and placed in RNAlater Solution (Ambion) for subsequent extraction of RNA. Three conditions were isolated: embryos injected with *NvFGFa1* MO, embryos injected with *NvFGFa2* MO and non-injected embryos for control. Total RNA was then extracted with the RNAqueous kit (Ambion), following the manufacturer’s protocol, and temporarily stored at −80 °C, in order to accumulate the quantity necessary for the hybridization (20 µg of total RNA for each condition). The microarray analysis was performed by Nimblegen (Roche), on a custom designed 385k cDNA chip based on the annotation of the *Nematostella* genome (Putnam et al., 2007) provided by the Department of Energy Joint Genome Institute (JGI, http://genome.jgi-psf.org/Nemve1/Nemve1.info.html). The probes used for detection were 60mer oligos, and (when possible) 7 of them were used per target. The array hybridizations were conducted in duplicate (technical). For normalization, the raw data (.pair file) was subjected to Robust Multi-Array Analysis (Irizarry et al., 2003), quantile normalization (Bolstad et al., 2003), and background correction as implemented in the NimbleScan software package (Roche NimbleGen, Inc.). Data were analysed with the J-Express 2009 software (Dysvik and Jonassen, 2001; Stavrum et al., 2008). Genes were selected on the basis of expression level fold change, when comparing control to injected conditions. The ao numbers (for apical organ) in this study are based on the 198 gene list identified by fold-change, not on the list of genes confirmed by in situ hybridization. Microarray data are deposited in ArrayExpress with accession number E-MTAB-3004.

### *Nematostella* in situ hybridization

Genes of interest obtained from the microarray were confirmed by in situ hybridization, performed as previously described (Rentzsch et al., 2008), in order to build a working dataset of apical organ genes.

### Identification and in situ hybridization of putative sea urchin orthologs

The genes that were confirmed to be expressed in the *Nematostella* apical organ were used as query sequences for an orthology search in *Strongylocentrotus purpuratus*, based on an integration of a reciprocal BLAST method (Wall et al., 2003) and best BLAST hit with sequences obtained from the SpBase website version 2.1 (http://www.spbase.org) and NCBI. The putative orthologous genes were investigated in sea urchin by in situ hybridization (Minokawa et al., 2004).

Pictures were taken with Nikon Eclipse E800 and a Nikon AZ100M microscope, and processed with Adobe Photoshop CS5.

### Identification of possible ciliary genes

**Fig. S1:**
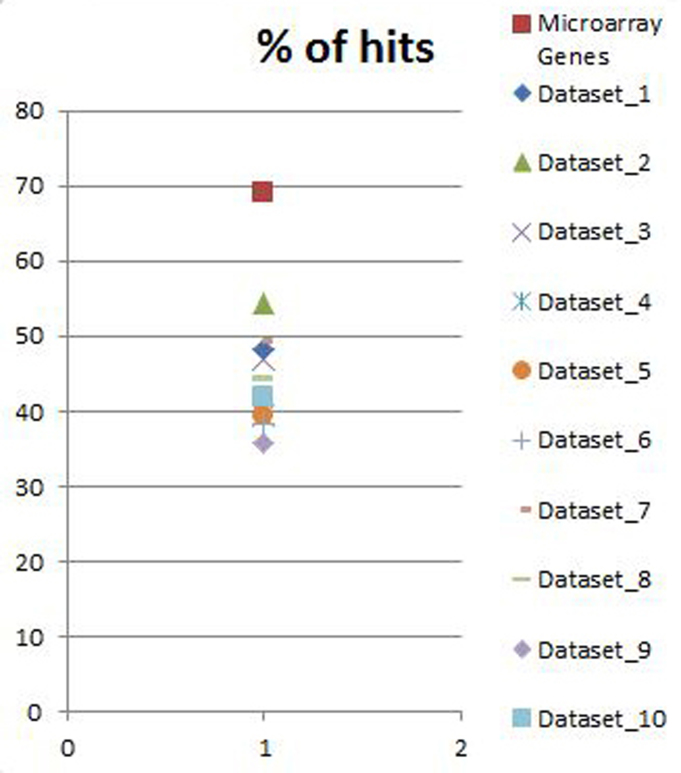
**The AO gene set is enriched for ciliary genes.** The percentage of BLAST hits against the custom ciliary database (Supplementary File 1) is shown for the AO dataset (red square) and for 10 random *Nematostella* datasets. The values of the random datasets are lower than the value for the AO dataset.

AO dataset genes potentially related to cilia were identified by an alignment search against an assembled database of ciliary genes, obtained from the Cildb v2.1 database (Arnaiz et al., 2009). The assembled database is provided in Supplementary File 1. By using a Standalone BLAST obtained from NCBI, the AO dataset was blasted against the ciliary database, using a threshold *e*-value of 1*e*^−5^. To validate the results, a randomization search was performed: 10 random datasets, the same number of genes as the AO dataset, were generated from the *Nematostella* genome and blasted against the ciliary dataset using the same parameters. Results are provided in Supplementary Fig. 1.

## Results

### Analysis of microarray data

The aim of the microarray analysis was to identify genes specifically expressed or enriched in the apical organ of *Nematostella*. The microarray was based on the annotated *Nematostella* genome and was used to compare the transcription profiles of three different conditions: a wild type situation with a properly formed apical organ, and two morphant situations, one with an expanded apical organ (*NvFGFa2* MO) and one without an apical organ (*NvFGFa1* MO, see Fig. 1C). The expression data were first evaluated for quality and reliability by analysing the expression profiles of previously described apical organ genes, e.g. *NvCOE, NvFGFa1*, *NvHoxF/Anthox1* (Finnerty et al., 2004; Pang et al., 2004; Rentzsch et al., 2008). This step confirmed the consistent up-regulation of apical organ genes in *NvFGFa2* MO injected animals, but revealed that these genes were not reliably down-regulated in the *NvFGFa1* MO injected animals. This might be due to the relatively small size of the apical organ in wild type planulae, which likely precludes the identification of significant gene expression changes when compared to planulae lacking the apical organ. In addition, non-specific effects of the *NvFGFa1* MO cannot be excluded. Therefore, the analyses were focused on the genes enriched in the expanded apical organ condition (when compared to wild type or no-apical-organ phenotype).

The selection of genes specifically up-regulated in the *NvFGFa2* MO condition, i.e. the possible candidates for apical organ specific genes, was based on a fold change criterion. The expression profiles of known apical organ genes were used to select a cut-off value of 1.8 fold change; for sequence-specific transcription factors this value was lowered to 1.5 fold.

With this approach, a set of 198 putative apical-organ (ao) genes was recovered. Classification by KOG terms (euKaryotic Orthologous Groups), which identify a possible biological function of the gene based on sequence similarities, was used for a first evaluation of the dataset (Table 1). Similar to the complete genome annotation, 49.6% of the recovered genes had no clear KOG assignment (48.1% for whole genome) and of these 26.3% were totally uncharacterized (27% for whole genome). Some KOG classes of genes are particularly over-represented in the AO dataset (at least 25% increase): (i) cell motility, (ii) nucleotide transport and metabolism, (iii) secondary metabolites biosynthesis, transport and catabolism, (iv) cytoskeleton, (v) intracellular trafficking, secretion, and vesicular transport, (vi) signal transduction mechanisms.

Even though the microarray hybridizations were performed with only two technical replicates and therefore could not be analysed statistically, the over-represented classes of genes are consistent with previous descriptions of apical organ structure, i.e. being composed of ciliated cells, rich in vesicles, and with a possible sensory and/or secretory function.

### A working dataset of apical-organ specific genes

To confirm the expression of the AO dataset in the apical organ region in situ hybridization was performed for 100 genes that are evolutionarily conserved as indicated by BLAST hits with scores >50 in other metazoan genomes. This systematic analysis showed that 78 genes were exclusively or predominantly expressed in the apical organ, while the remaining genes either had no detectable expression or weak uniform expression. A list of the newly identified apical organ genes is provided in Table 2, expression patterns for all 78 genes at gastrula and planula stages are shown in Fig. S2.

This confirmed dataset of 78 genes constitutes the working set of AO genes, which were used in the subsequent analyses. The recovered genes are very diverse, including signalling pathway components, transmembrane molecules, transcription factors, and many uncharacterized genes.

### Identification of cilia-related genes, up-regulated in the apical organ

As described in the introduction, the major discernible feature of the *Nematostella* apical organ is the tuft of long cilia, and long cilia are also a general distinctive trait of bilaterian apical organs. While different types of cilia share a core of some structural components, they differ with respect to other structural proteins, metabolic proteins, their developmental program and signalling factors (Choksi et al., 2014; Silverman and Leroux, 2009; Takeda and Narita, 2012). The molecular composition of these cilia could thus be informative for the comparison of apical organs in different taxa. However, whether apical organ cilia have molecular characteristics that distinguish them from other cilia is currently unclear.

Due to the wide array of diseases caused by defects in cilia development or function, many studies have contributed to the generation of databases of genes and proteins involved in ciliary processes (Arnaiz et al., 2009; Gherman et al., 2006; Pazour et al., 2005). These databases and the rich literature allowed an in-depth search for putative ciliary genes within the apical organ dataset. For this purpose a dataset was assembled from ten species in which high throughput ciliary studies have been performed, including vertebrates (*Homo sapiens, Rattus norvegicus, Mus musculus, Xenopus laevis*), protostomes (*Drosophila melanogaster, C. elegans*), protozoans (*Trypanosoma brucei,* and the ciliates *Paramecium tetraurelia, Tetrahymena thermophila)* and the green alga *Chlamydomonas reinhardtii.* The analysis of the *Nematostella* AO dataset against this multi-species ciliary database (Supplementary File 1, see also the Materials and methods section) indicated that the AO dataset is indeed enriched in ciliary genes (Fig. S1). Specifically, it identified a possible cilia-related function for 52 genes of the working dataset (see Table 2), however, this does not exclude the possibility that these genes are also involved in additional, non-ciliary cellular processes.

**Fig. 2:**
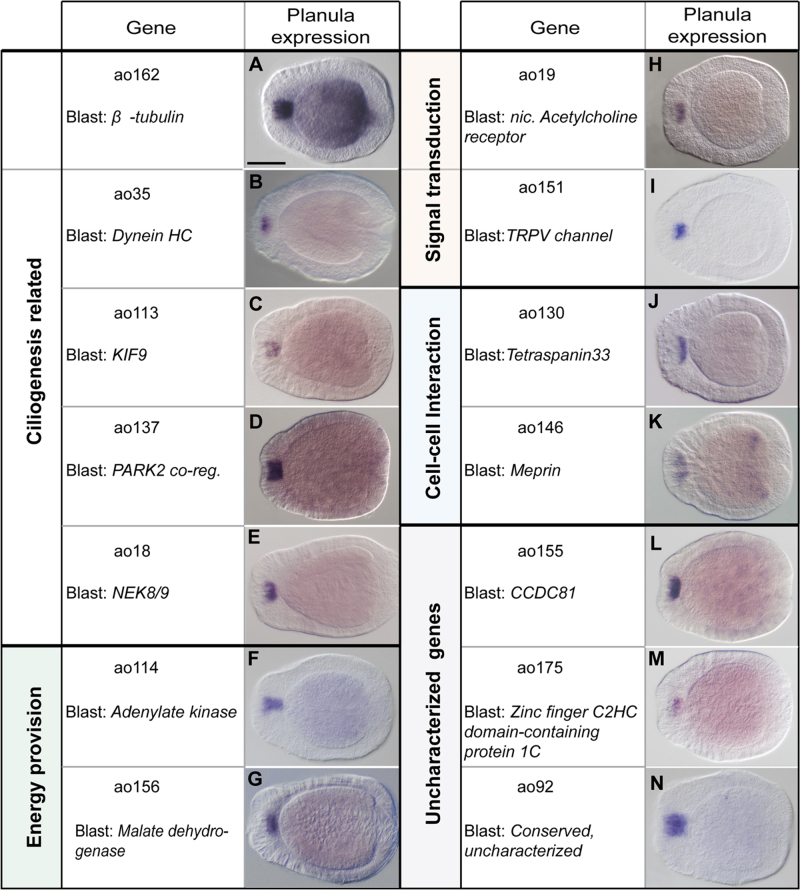
Examples of new apical organ genes related to cilia development and function A BLAST search against a custom dataset of cilia-related genes allowed the identification of 52 putative ciliary genes. These included genes that are related to general aspects of ciliogenesis (A–E), like a *β-tubulin* gene (A), and genes which might give a clue about the nature of the apical organ cilia, being related to provision of energy (F and G), transduction of signals (H and I) and cell–cell interactions (J and K). Interesting is also a number of conserved but uncharacterized genes, here identified with the putative orthologous human gene (L–N). The embryos displayed are all at planula stage, the aboral pole is to the left. Each gene is identified by the assigned ID and a name, either attributed by the genome annotation, or obtained through a BLAST search. Scale bar=100 µm.

The 52 putative cilia-related genes can be considered in several categories. The first category includes genes involved in ciliogenesis. The forkhead domain containing gene *NvFoxJ1(ao194)* is among the few transcription factors that were recovered in the analysis. *FoxJ1* has been characterized as a key regulator of the development of motile cilia in vertebrates and this function has been suggested to be conserved across metazoans (Thomas et al., 2010; Vij et al., 2012). The onset of *NvFoxJ1* expression at the aboral pole of *Nematostella* coincides with the differentiation of the apical organ at early planula stage (Sinigaglia et al., 2013). The assembly and maintenance of cilia depends on conserved structural proteins and an intraflagellar transport (IFT) system. Genes falling into this category are *Nvβ-tubulin (ao162)*, *NvTektin (ao90)*, *NvPACRG*-*like* (*Parkin-co-regulared gene (ao101, ao137;* (Dawe et al., 2005), and the motor protein components *NvDynein heavy chain-like (ao35)* and *NvDynein light chain-like (ao77)* (Fig. 2A–D). Kinesins are microtubule-binding motor proteins that have diverse cellular functions including intraflagellar transport (Hirokawa et al., 2009; Verhey et al., 2011). Two *Nematostella* kinesins, *NvKif9-like* (*ao113*) and *NvKif16-like (ao74)*, are expressed in the apical organ; however, members of these particular kinesin families have not been implicated in intraflagellar transport (see the Discussion section).

Nima-related kinases 1 and 8 (NEK1 and 8) are associated with the cilium in mammals and in *Chlamydomonas* and they have been implicated in vertebrate ciliopathies (Bradley and Quarmby, 2005; Liu et al., 2002; Upadhya et al., 2000). Their exact function is not known and it is unclear whether they are restricted to particular subsets of cilia. In *Nematostella*, a *NEK8/9-like* (*ao18*) gene is expressed in the apical organ from early planula stage on (Fig. 2E).

Cilia constitute a separate compartment of the cell, distinct from the rest of the cytoplasm (Nachury et al., 2010). However, they require a constant provision of energy for mobility and to maintain the activity of the intraflagellar transport machinery (Rosenbaum and Witman, 2002). This high demand for energy is supported by metabolic enzymes located in the cilium, as demonstrated in mammals and *Chlamydomonas* (Mitchell et al., 2005; Mukai and Okuno, 2004). Several genes involved in energy provision were recovered in the screen. For example, adenylate kinase (ADK) regenerates ATP from ADP and has been shown to localize and function in cilia in various organisms (Fernandez-Gonzalez et al., 2009; Nakamura et al., 1999). Two *Nematostella ADK-like* genes (*NvADK-like 1* and *2, ao49* and *ao114*) were found to be expressed in the apical organ (Fig. 2F and Fig. S2PA)). There are also genes coding for enzymes involved in metabolic pathways, in particular enzymes producing NADH+H like malate dehydrogenase (*NvMDH, ao156*) and aldehyde dehydrogenase (*ao171*) (Fig. 2G).

Several studies have provided evidence that not only immotile, but also motile cilia can have sensory functions, as for example in the case of the motile cilia of the respiratory epithelium of mammals, which sense mechanical and chemical cues (Johnson et al., 1991; Shah et al., 2009). Similarly, our screen revealed the presence of genes with a possible function in the sensing and transduction of signals. A direct connection between the apical organ of *Nematostella* and the nervous system has not been shown (Nakanishi et al., 2012), but two genes coding for nicotinic acetylcholine receptors (*ao19* and *ao145*) are expressed in the apical organ cells (Fig. 2H and Fig. S2) and may mediate signalling between the apical organ and the underlying plexus of neurites. Another gene potentially involved in a sensory function is *NvTRPV-like (ao151;* Fig. 2I**)**, which is related to calcium channels of the Transient Receptor Potential (TRP) family. Members of this group of ion channels are involved in mechano-, chemo- and thermo- sensation (Liedtke and Kim, 2005) and interestingly, a *TRPV* gene is expressed in the apical tuft cells of *P. dumerilii* (Marlow et al., 2014).

Other genes that are predicted to localize to the plasma membrane or to the extracellular matrix (ECM) and are thus potentially involved in cell–cell or cell-ECM interactions include the transmembrane protein *NvTetraspanin33-like* (*ao130,* Fig. 2J), the astacin metalloprotease *NvMeprin-like (ao146,* Fig. 2K) (Sterchi et al., 2008), and the ECM protein *NvSpondin1-like* (*ao147,* Fig. S2AB) (Hemler, 2005).

Nearly half of the potentially cilia-related apical organ genes are poorly characterized. In some cases a specific domain is recognizable, for example in the coiled-coil domain containing genes *NvCCDC74-like (ao60)*, *NvCCDC81-like* (*ao155*) and *NvCCDC121-like (ao95)* or in three Leucine-Rich Repeat containing genes (*ao43*, *ao46*, *ao62*; Fig. 2L–N). However, several other genes display sequence conservation but lack recognizable domains (Table 2).

### “Non-ciliary” genes

As for the cilia-related genes, about half of the apical organ genes that are not found in the ciliary databases are evolutionarily conserved, but uncharacterized.

**Fig. 3:**
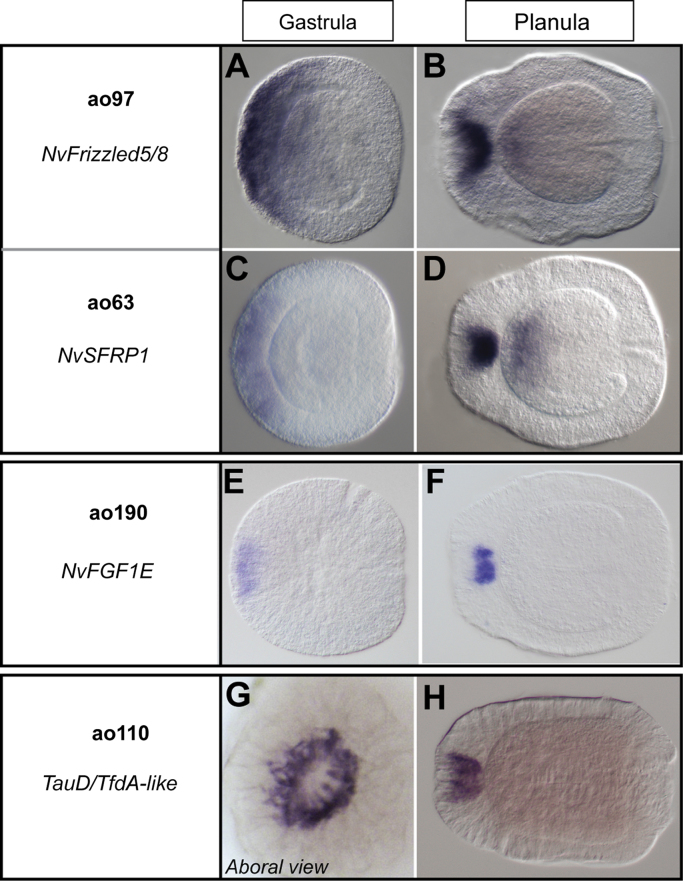
Selected non-ciliary apical organ genes The genes which did not produce any hit against the custom dataset of cilia-related genes are considered as “non-ciliary”. (A-D) Two genes involved in the Wnt signalling pathway were recovered, the Wnt receptor *NvFz5/8* (A and B) and the secreted protein *NvSRFP1* (C and D). Both genes are expressed in a broader aboral domain at gastrula stage (A and C), then restrict to the most aboral pole of the planula. Aboral endodermal expression is also visible at this stage. (E and F) *NvFGF1E* is expressed in a relatively small aboral domain at gastrula stage and in a subset of apical organ cells at planula stage. (G and H) A gene orthologous to the *E. coli TauD* gene was also found. The gene is involved in the catabolism of taurine, an amino acid that has been implicated in metamorphosis. *NvTauD* is expressed in a ring within the apical organ domain (see aboral view of the planula, in E), demonstrating the existence of different sub-domains within the apical organ domain. The aboral pole is to the left, each gene is identified by the assigned ID and a name, either attributed by the genome annotation, or obtained through a BLAST search. Scale bar=100 µm.

Regarding the characterized genes, the non-ciliary dataset contained two genes related to the Wnt signalling pathway, which determines the site of gastrulation in bilaterians and cnidarians and is involved in the patterning of the anterior–posterior axis of bilaterians. Both the Wnt receptor *NvFrizzled5/8* (*ao97*) and the putative Wnt antagonist secreted Frizzled-Related protein (*NvSFRP1, ao63*) are expressed in a broad aboral domain at gastrulation and at highest levels in the apical organ of the planula larvae (Fig. 3A–D**)**, and (Kumburegama et al., 2011)). While the function of the two Fibroblast growth factor genes *NvFGFa1* and *NvFGFa2* in apical organ development has been described (Rentzsch et al., 2008; Sinigaglia et al., 2013), there are 11 *Nematostella* FGFs for which expression data are not yet available. We identified *NvFGF1e (ao190)* (Matus et al., 2007) as an additional apical organ related FGF. *NvFGF1e* is expressed at the aboral pole from gastrula stage on and its expression at planula stage is restricted to a subset of apical organ cells (Fig. 3E and F).

Among the metabolic genes, we recovered a gene for the catabolism of the amino acid taurine, a homolog of the *TauD/Tfd*A (*ao110*) gene of *E. coli* (Fig. 3G and H). Taurine is involved in a wide array of biological functions such as osmoregulation, antioxidation, modulation of neurotransmitters, stimulation of glycolysis, and maintenance of photoreceptors (Huxtable, 1992). In particular, in marine invertebrates taurine is important for the development and settlement of larvae, and in cnidarian larvae it is probably functioning as an inhibitor of metamorphosis (Berking, 1988), but see also (Walther, 2002). The expression of the *NvTauD* gene in *Nematostella* could therefore support a role for the apical organ in metamorphosis, as previously suggested. Indeed, *Nematostella* larvae lacking an apical organ do not enter metamorphosis, as demonstrated by the *NvFGFa1* MO injected animals (Rentzsch et al., 2008).

### Genes expressed in the apical organ and in specific cell-types

**Fig. 4:**
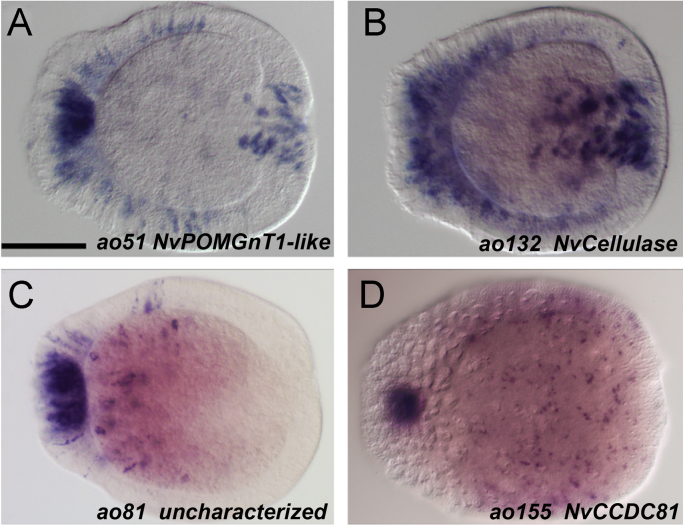
Apical organ genes with additional cell-type specific expression (A) The O-linked-mannose beta-1,2-*N*-acetylglucosaminyltransferase gene *NvPOMGnT1-like* (*ao51*) is expressed in scattered cells in the ectoderm and in the pharynx. (B) *NvCellulase* positive cells (*ao13*2) are enriched in a broad domain in the aboral ectoderm and in the pharynx, scattered ectodermal cells are also present. (C) The uncharacterized gene identified by the ID number 239479 (*ao81*) is expressed in few ectodermal cells in the aboral half of the larvae. (D) The coiled-coil domain containing gene *NvCCDC81* (*ao155*) is detected in individual cells throughout the entire ectoderm (picture focuses on the surface). The embryos displayed are all at planula stage, the aboral pole is to the left. Scale bar=100 µm.

Some of the identified apical organ genes displayed additional expression that appeared to be restricted to particular cell-types. The O-linked-mannose beta-1,2-*N*-acetylglucosaminyltransferase gene *NvPOMGnT1-like* (*ao51*) is expressed in scattered cells in the ectoderm and in the pharynx (Fig. 4A). *NvCellulase*-positive cells (*ao132*) are enriched in a broad domain in the aboral ectoderm and in the pharynx (Fig. 4B). Compared to the often spindle-shaped *NvPOMGnT1-like* expressing cells, the *NvCellulase*-positive cells appear more compact. On the basis of the predicted gene function and the distribution of these cells, we assume that the *NvCellulase*-positive cells are a particular type of gland cells (gland cells with translucent vesicles (Nakanishi et al., 2012)). One of the uncharacterized apical organ genes (*ao81*) is expressed in only a few ectodermal cells in the aboral half of the larvae, whereas the coiled-coil domain containing gene *NvCCDC81 (ao155)* can be detected in individual cells throughout the ectoderm (Fig. 4C and D).

### A dataset for evolutionary comparisons: Sea urchin

The main goal of this study was to establish an improved basis for the comparison of cnidarian and bilaterian apical organs, which could allow a better understanding of their evolutionary histories.

As a test case for an initial comparative study we chose the purple sea urchin *S. purpuratus*, as it is an established model system for developmental studies with a sequenced genome and with an apical organ at embryonic stages. The development of the sea urchin apical territory has been addressed by different studies (reviewed in Angerer et al., 2011), and a recent paper showed similarities with the determination of the apical territory in *Nematostella* (Sinigaglia et al., 2013). The sea urchin larva has two prominent ectodermal structures with long cilia: the apical organ and the ciliary bands. The presence of a second structure with long cilia could help to distinguish between genes that are generically involved in cilia formation and whose expression level simply reflects the length of the cilia, and genes that are specific to the apical organ tuft.

**Fig. 5:**
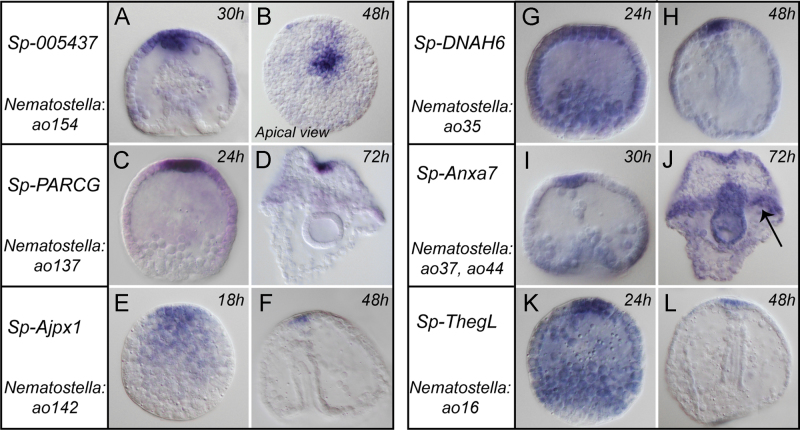
Expression patterns displayed by AO homologous genes in the purple sea urchin. Different stages are displayed, to exemplify the expression dynamics: genes could be either restricted to the apical organ (e.g. A–D) or be broadly expressed in the early stages and then restricted to the apical organ (E–H, K, L), or display additional domains like in the ciliary bands (I and J, arrow indicates ciliary band). Pictures are lateral views (except B), oriented with the blastopore at the bottom. Each gene is identified with the name obtained from the sea urchin database at www.spbase.org; the corresponding *Nematostella* gene is also reported.

The orthology search identified 73 putative sea urchin genes homologous to the genes in the *Nematostella* AO dataset, of which several have previously been shown to be expressed in the apical territory (e.g. *frizzled 5/8, SFRP1, beta tubulin, foxJ1, Tektin* (Croce et al., 2006a; Dunn et al., 2007; Illies et al., 2002; Poustka et al., 2007; Tu et al., 2006)). For an initial in situ hybridisation analysis 18 genes were selected for which no spatial expression data were available. Twelve of the 18 genes showed specific or enriched apical organ expression (Fig. 5 and Fig. S3) sometime during the developments of the sea urchin larva, while for the others no expression was detected (with the exception of one gene expressed in the gut, *Aldh2*). Of the genes with apical organ expression some were specifically expressed in the apical organ area (e.g. *PACRG*, Fig. 5C and D), while others had later additional domains in the ciliary band and few in the ciliated gut (e.g. *Annexin*, Fig. 5I and J). The gene for an acetylcholine receptor had a patchy ectodermal expression domain, with an enrichment of cells in the apical organ (Fig. S3).

## Discussion

In this study we have identified a set of genes that are expressed in the apical organ of the anthozoan *N. vectensis*, providing a molecular signature for the apical organ cells. The recovered genes are very diverse and constitute a new and versatile tool for developmental, functional and comparative studies of apical organs across metazoans. However, only seven out of 15 previously described apical organ genes were recovered with the cut-off values chosen for the initial analysis (the 198 gene list, see Table S1) and our dataset therefore likely represents an underestimation of all apical organ specific or enriched genes.

### A signature for “apical organ” cells

Though a bundle of long cilia is considered the hallmark of apical organs both in bilaterian and cnidarian larvae, these aboral cilia can be more or less prominent in different larvae, in particular among cnidarians (e.g. Widersten, 1968). It is also not clear whether the long cilia are a prerequisite for the function of apical organs, or if functionally equivalent apical organ-like sensory structures with short cilia exist. For example, the morphology and cell type composition of the aboral pole of planulae of the scyphozoan cnidarian *Aurelia* is highly similar to the apical organ region of anthozoan planulae, but it lacks the prominent tuft of long cilia (Chia and Koss, 1979; Nakanishi et al., 2012, 2008; Yuan et al., 2008). Interestingly, the apical organ-like region of *Aurelia* contains taurine immunoreactive sensory cells (Nakanishi et al., 2008) and *NvTauD*, a gene involved in taurine catabolism, is expressed in the apical organ region of *Nematostella* (this study). The essential role played by cells localized in the aboral part of the larvae has been described also in several coral species, such as *Pocillopora damicornis,* where each sensory cell bears a single cilium surrounded by a collar of microvilli (Tran and Hadfield, 2013; Vandermeulen, 1974), or in *Acropora* species, in which the aboral pole has been shown to have a role in the recruitment to the substrate (Okubo and Motokawa, 2007). Given that the settlement of coral larvae is a highly specialized process with significant impact on reef communities (see for example Morse et al., 1988), a deeper understanding of the molecular processes and of the cell types involved is of ecological importance. Our dataset could therefore help in identifying “apical organ” cell types, even in those cases in which a prototypical apical organ, with distinctive long cilia, is missing.

### Molecular characteristics of apical tuft cilia

An open question about the long apical organ cilia is whether they are molecularly distinct from the shorter cilia that cover other parts of the larval epidermis. Cilia are highly conserved structures of eukaryotic cells, with fundamental roles in locomotion, movement of fluid or in the sensing of external cues (for a recent review see Choksi et al., 2014). The core element of a cilium is the axoneme, consisting of nine doublets of microtubules emerging from the basal body, and surrounded by a membrane in continuity with the plasma membrane of the cell. Cilia are usually classified in two types according to their axonemal architecture: the 9+0 type cilia are sensory and immotile, while the 9+2 type cilia (with two central microtubule singlets) are considered as motile. However, there are many exceptions to this classification, and recent studies have suggested that all cilia could have a sensory role (Bloodgood, 2010). The majority of the conserved genes that we identified as enriched or specific to the apical organ of *Nematostella* are associated with the development, structure or function of cilia, although they can have additional, non-ciliary functions. While several classes of structural and motor proteins are required in all cilia, the evolutionary diversification of some of these components makes them informative for the comparison of apical organ cilia and other cilia. For example, at least eleven distinct families of Kinesin motor proteins were present in the last common ancestor of eukaryotes and 45 kinesin genes have been identified in mammals (Miki et al., 2005; Wickstead et al., 2010). Kinesins are involved in several intracellular processes and this is partially reflected in specific expression patterns (Hirokawa et al., 2010, 2009; Mazumdar and Misteli, 2005). Accordingly, the expression of *NvKif9-like* may reflect a specific function in apical organ cilia. *NvKif9-like* belongs to the Kinesin 9 family which is poorly studied, but the *Chlamydomonas* KIF9 protein Klp1 is unusual in that it localizes to the central microtubules of the cilium and is required for ciliary motility (Yokoyama et al., 2004). *Kif16* genes have not been related to ciliary functions. Instead, *Kif16b* has been shown to regulate endosome trafficking, including the transport of the FGF receptor to the cell membrane (Hoepfner et al., 2005; Ueno et al., 2011), whereas the Kif16a protein localizes to centrosomes and has been implicated in mitotic spindle formation (Torres et al., 2011). Thus, the expression of *NvKif16-like* in the apical organ may not be related to the development or function of cilia.

A *caveat* for the interpretation of our data is that in situ hybridization cannot rule out a uniformly low level of expression of the identified genes in the ciliated ectoderm. This means that the strong expression in the apical organ cells might only reflect the length of the apical organ cilia and a correspondingly higher demand for the gene products in these cells. The analysis of larvae with ciliary bands will be particularly informative in addressing this problem. If the expression level of a particular gene is relative to the length of the cilia, then ciliary bands would be expected to display stronger signal than the remaining ectoderm. Indeed, while expression of the *Park2 co-regulated gene* (*PACRG*) in sea urchin is restricted to the apical pole, another homolog of a *Nematostella* apical organ gene, *AnnexinA*, is expressed in the apical organ and in the ciliary bands (see also below).

### Apical organ-specific developmental regulators

Transcription factors and signal transduction pathways regulate the development of body regions, organs and cell types and are frequently employed to address questions of homology. In this study we identified relatively few developmental regulators, probably due to the often comparably low expression level of these genes, which can hinder their identification in microarray experiments with low replicate numbers. Among the developmental genes that we found were two Wnt pathway components, the Wnt receptor *NvFrizzled5/8*, a putative positive regulator of the pathway, and *NvSFRP1*, a putative negative regulator. Both genes have been shown to be expressed at the anterior pole of some bilaterian larvae or embryos, e.g. in sea urchin, in the hemichordate *Saccoglossus kowalevskii* and the annelid *P. dumerilii* (Croce et al., 2006b; Darras et al., 2011; Illies et al., 2002; Marlow et al., 2014; Pani et al., 2012). In bilaterians and cnidarians, Wnt signalling is required for the determination of the gastrulation site and for the patterning of the apical-blastoporal axis. However, the regulation by NvFGF signalling suggests that in addition to an early patterning function, *NvFrizzled5/8* and *NvSFRP1* may have separate, later functions in the development of the apical organ.

Of the transcription factors that we identified, *NvFoxJ1* is of particular interest. *FoxJ1* is a key regulator of motile cilia formation in vertebrates and it has been suggested to be a regulator of this process across the Metazoa (Choksi et al., 2014; Stubbs et al., 2008; Thomas et al., 2010; Vij et al., 2012; Yu et al., 2008). In vertebrates, *foxJ1* has been shown to be regulated by FGF and Wnt signalling (Caron et al., 2012; Neugebauer et al., 2009), a situation which might also be the case in *Nematostella* (see also Sinigaglia et al., 2013). The strong expression of *NvFoxJ1* in the apical organ together with the expression of genes that have been associated with the high demand for energy of motile cilia (*NvADK-like, NvMDH*) indicates that the long cilia of the *Nematostella* apical organ are motile cilia. Functional studies will have to determine whether *NvFoxJ1* is specifically required for the development of the apical organ cilia or whether the motile cilia of the body surface also depend on the function of this gene.

Overall, functional studies of the developmental regulators identified in this study will help to characterize the developmental programs of apical organ cells in *Nematostella* and to compare it to other organisms, but their relatively small number does not allow a comprehensive understanding of apical organ formation and evolution.

### Comparison to the apical organ of sea urchins

Our experimental approach aimed at the identification of apical organ genes as a versatile tool for studying apical organs in different animal groups. The analysis of a subset of the identified genes in the developing larvae of the sea urchin *S. purpuratus* supports the suitability of this approach. In particular, we could discriminate between genes expressed solely in the apical organ (*Sp-Ajpx1*), or with additional domains in the ciliary bands (*Sp-Anxa7*) or even specific to particular ciliated territories, such in the case of the *Aldh* gene, which is expressed in the apical organ and the endoderm of *Nematostella* and in the mid-portion of the gut of sea urchin larvae (Fig. S2QB and S3). Furthermore, the usability of the dataset to describe conserved aspects of previously uncharacterized genes is demonstrated by the expression of Sp-005437 (Fig. 5A and B; most similar to *Nematostella ao154*) in the apical domain of the sea urchin. This gene encodes a short predicted protein (83 and 68 amino acids in sea urchin and *Nematostella*, respectively) that lacks annotated domains and is also conserved in the hemichordate *S. kowalewskii*.

In conclusion, we have identified a set of 78 new apical organ genes that are mainly related to structural and physiological aspects of the apical organ cells. These genes serve as an entry point for a better understanding of the structure and function of the apical organ of *Nematostella* and they enable comparisons of the molecular composition of apical organs within Cnidaria, and in different bilaterian taxa, as exemplified by the expression analysis of a subset of these genes in the sea urchin. Therefore, the results of this study provide additional characters to address the homology of apical organ cell types, but they also serve as a generic tool to identify aboral cells that may play a role in the sensing of environmental cues.

## Figures and Tables

**Fig. S2-1:**
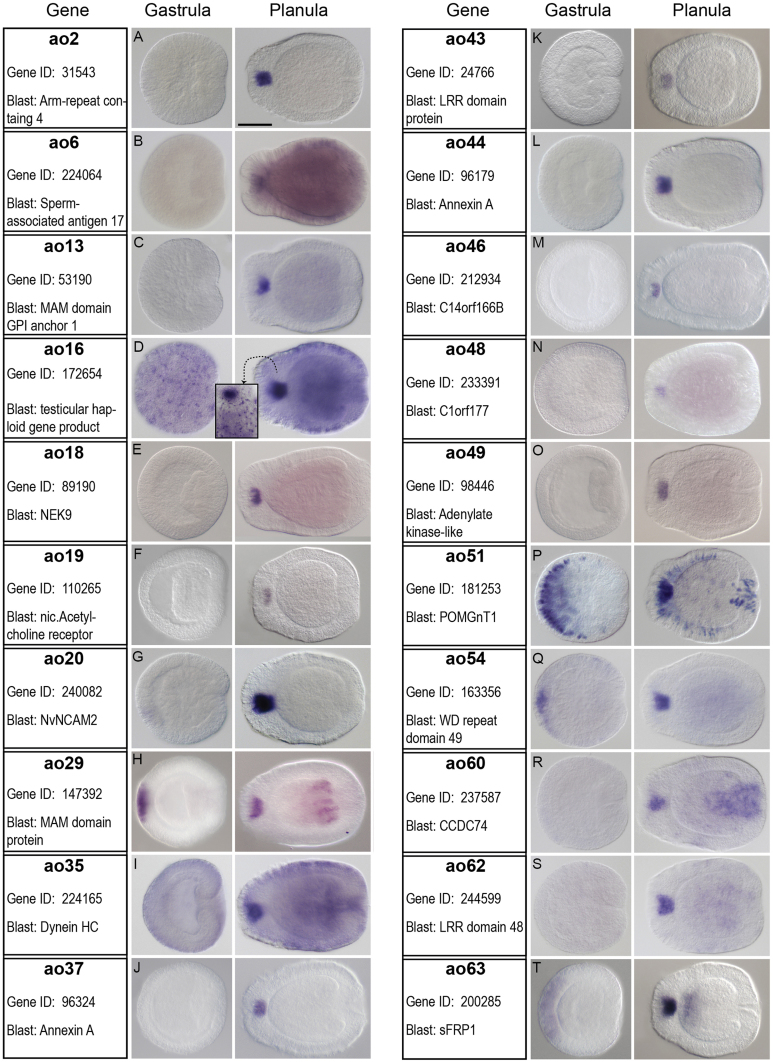
**Expression patterns of*****Nematostella*****apical organ genes.** In situ hybridizations at gastrula and planula stages for the genes identified in this study. All images are lateral views with aboral pole to the left. The insets are: (D) aboral view, (DA, AB, VA) higher magnifications. *Note*: some pictures are also shown in the main figures. Scale bar=100 µm

**Fig. S2-2:**
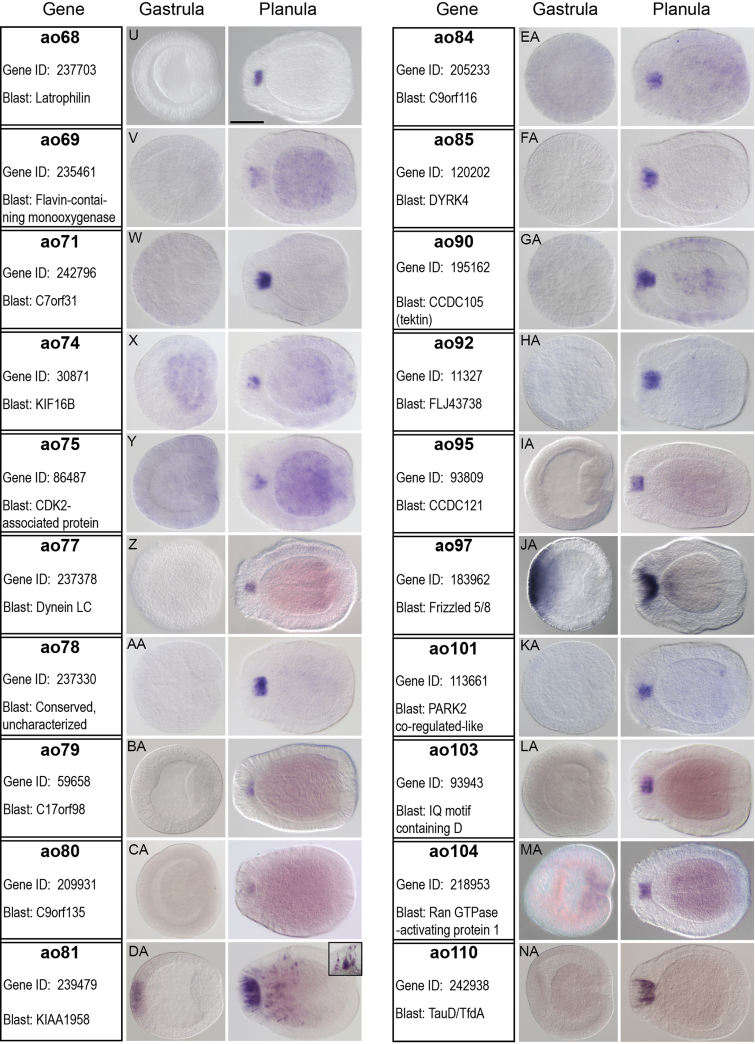


**Fig. S2-3:**
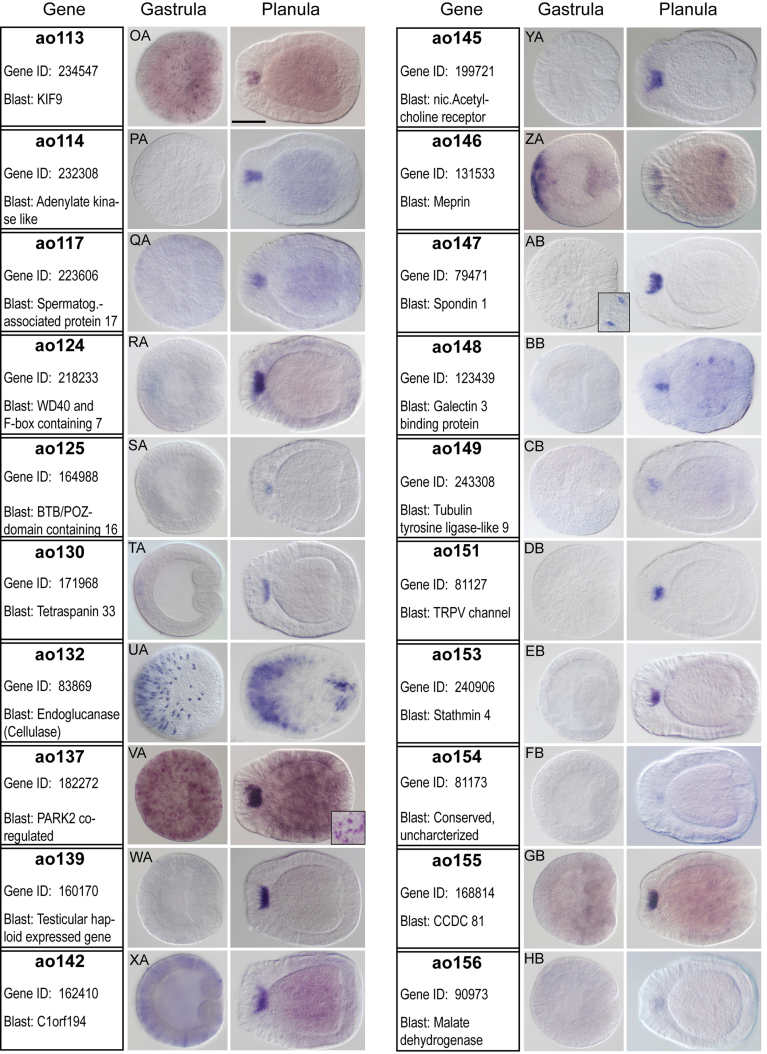


**Fig. S2-4:**
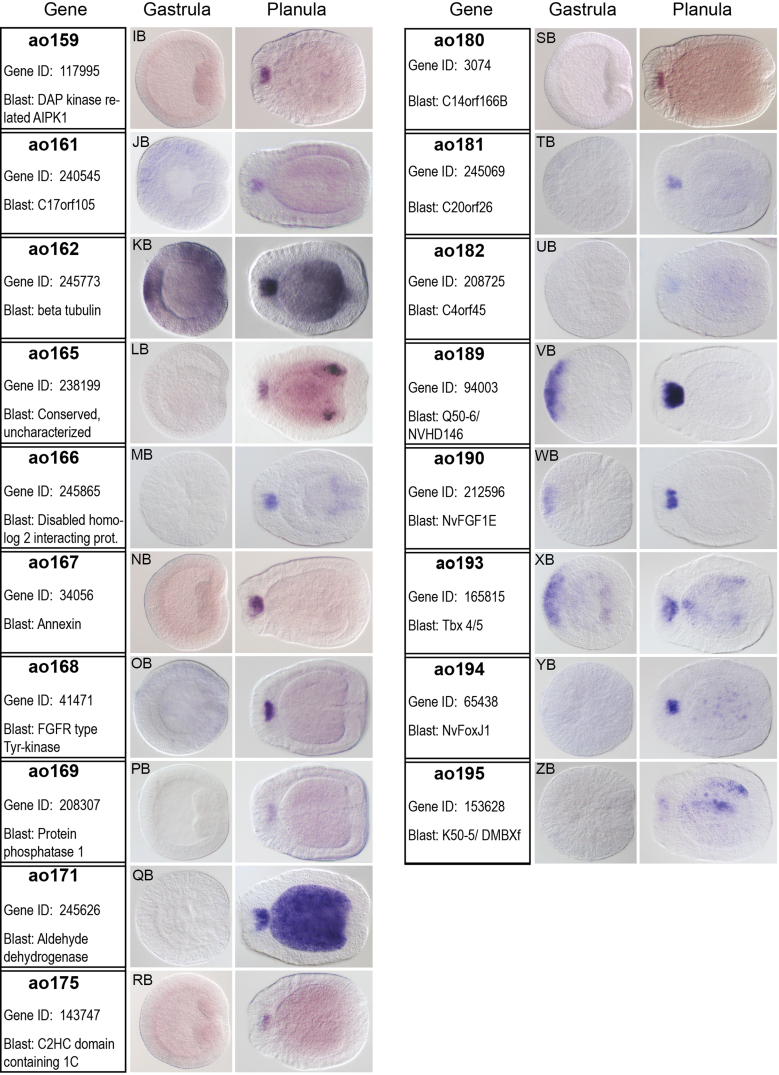


**Fig. S3-1:**
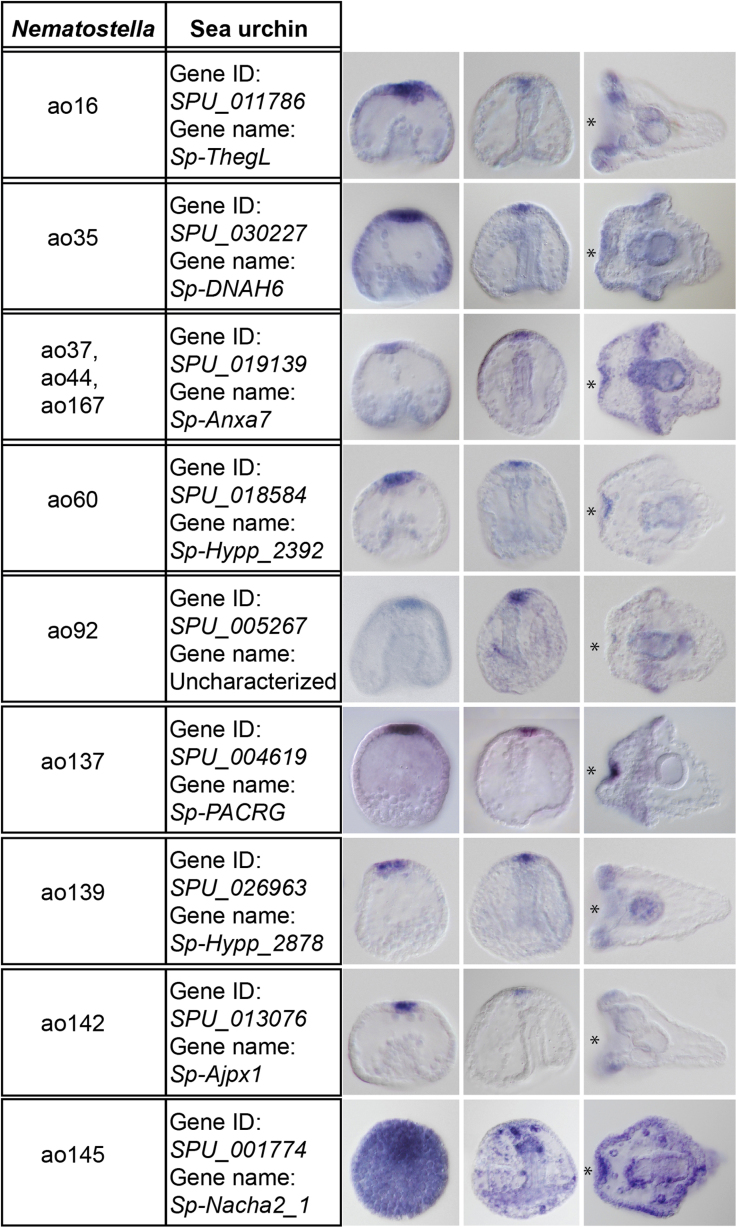
**Expression patterns of putative sea urchin apical organ genes.** In situ hybridizations for the sea urchin genes most similar to the indicated *Nematostella* genes (by reciprocal BLAST). For each gene from left to right: lateral views of mid-gastrula, late gastrula (with apical pole to the top) and pluteus (oral side indicated by asterisk) stages, respectively. For SPU_007284 an apical pole view is shown in the middle.

**Fig. S3-3:**
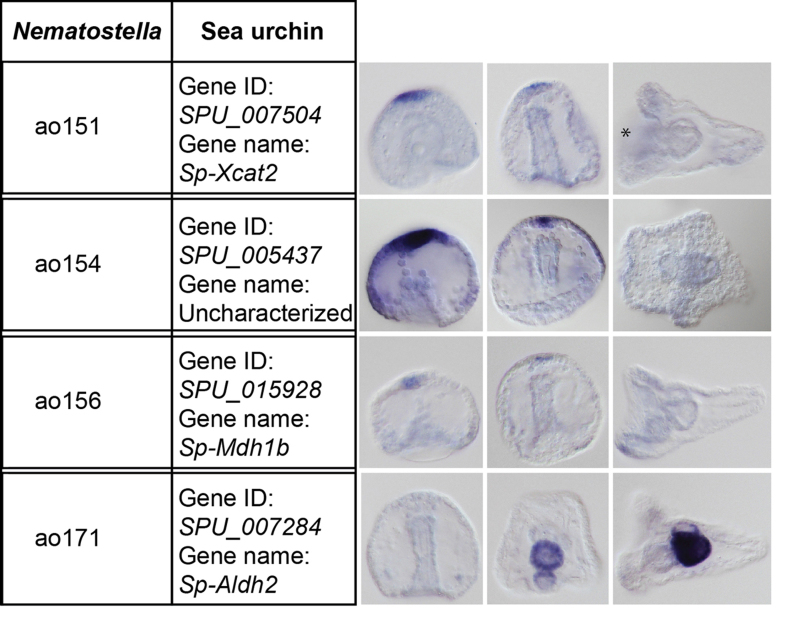


**Table 1:** **KOG class analysis of putative apical organ genes**. Relative abundances of the various KOG classes of genes, in the whole genome and in the condition with an expanded apical organ (*NvFGFa2* MO). In both cases, nearly half of the genes are poorly characterized (“*Function unknown*”, “*General function, prediction only*” and “*Uncharacterized- No KOG assigned*”). The last column indicates with the signs±the direction of the change, i.e. if a class is more or less abundant in the *NvFGFa2* MO injected when compared to the wild type (whole genome). The categories particularly overrepresented in the *NvFGFa2* MO condition are highlighted in red. The numbers displayed are percentages.

**KOG class**	***Nematostella*****genome (v 1.0)**	**Genes upregulated in*****NvFGFa2*****MO**	**Change compared to genome**
Cell motility	0.2	1.5	+
Defence mechanisms	0.3	0.0	−
Nuclear structure	0.3	0.5	+
Cell wall/membrane/envelope biogenesis	0.5	0	−
Coenzyme transport and metabolism	0.6	0	−
Nucleotide transport and metabolism	0.7	1.5	+
Cell cycle control, cell division, chromosome partitioning	1.0	0.5	−
Secondary metabolites biosynthesis, transport and catabolism	1.3	2.0	+
Replication, recombination and repair	1.3	0.5	−
Energy production and conversion	1.4	1.0	−
Chromatin structure and dynamics	1.4	1.0	−
Lipid transport and metabolism	1.7	0.0	−
Translation, ribosomal structure and biogenesis	1.8	0.5	−
RNA processing and modification	2.3	2.0	−
Carbohydrate transport and metabolism	2.3	1.5	−
Amino acid transport and metabolism	2.6	2.0	−
Extracellular structures	2.6	1.5	−
Inorganic ion transport and metabolism	2.7	2.5	−
Cytoskeleton	3.0	6.1	+
Intracellular trafficking, secretion, and vesicular transport	3.2	4.0	+
Transcription	3.6	3.5	−
Posttranslational modification, protein turnover, chaperones	6.0	4.0	−
Signal transduction mechanisms	11.1	14.1	+
Function unknown	5.5	7.1	+
General function prediction only	15.6	16.2	+
Uncharacterized (No KOG assigned)	27.0	26.3	−
**Total number of genes**	**25,911**	**198**	

**Table 2:** **List of the genes with confirmed apical organ expression**. This gene list constitutes the AO working list. Domain assignment was performed with SMART, including Pfam domains. For conserved, uncharacterized genes the best hit in the human genome is indicated. Note that some of these genes have no homologs in humans. Classification as ciliary or non-ciliary is based on the dataset provided in Supplementary File 1. The last two columns report the results of the search for putative orthologs in sea urchin. The first symbol (+ or −) indicates whether a putative ortholog was identified, the second whether it is expressed at the aboral pole (+) or not (−), (?) indicates no data avalaible.

**No in this study**	**Gene ID**	**KOG**	**KOG class**	**Domains (SMART+Pfam)**	**BLASTP result (provisional gene name)**	**Cilia-related (BLAST hit)**	**In sea urchin**	
							Putative ortholog/aboral expression	Putative ortholog gene ID
**2**	31543	KOG0167	Function unknown	Armadillo repeats	Arm-repeat containing 4, yeast vacuolar protein 8	X	+/?	SPU_007521

**6**	224064	*No KOG*	- - - - - - - -	*None*	Sperm associated antigen 17	X	+/?	SPU_013103

**13**	53190	KOG3627	Amino acid transport and metabolism	MAM (Meprin, A-5 protein, Mu)	MAM domain containing glycosylphosphatidylinositol anchor 1		+/?	SPU_015727

**16**	172654	*No KOG*	- - - - - - - -	THEG (Testicular Haploid Expressed Gene)	Testicular haploid expressed gene product		**+/+**	SPU_011786

**18**	89190	KOG1426	Function unknown	RCC1 (Regulator of Chromosome Condensation)	NEK9	X	+/?	SPU_019063

**19**	110265	KOG3645	Signal transduction mechanisms	Neurotransmitter-gated ion-channel	Acetylcholine receptor, alpha subunit	X	+/?	SPU_001774

**20**	240082	KOG0613	Cytoskeleton	Ig-like, FN III	NvNCAM2	X	+/?	SPU_005613

**29**	147392	KOG3627	Amino acid transport and metabolism	MAM (Meprin, A-5 protein, Mu)	MAM domain protein *(potentially part of ao146)*		+/?	SPU_004114

**35**	224165	KOG3595	Cytoskeleton	*None*	Dynein heavy chain, axonemal	X	**+/+**	SPU_030227

**37**	96324	KOG0819	Intracellular trafficking, secretion, and vesicular transport	Annexin	Annexin A	X	**+/+**	SPU_019139

**43**	24766	KOG4308	Function unknown	LRR	LRR protein	X	+/?	SPU_005282

**44**	96179	KOG0819	Intracellular trafficking, secretion, and vesicular transport	Annexin	Annexin A	X	**+/+**	SPU_019139

**46**	212934	KOG4308	Function unknown	LRR (Leucine-Rich Repeat), EF hand	*Conserved, uncharacterized* (C14orf166B)	X	+/?	SPU_005282

**48**	233391	*No KOG*	- - - - - - - -	*None*	*Conserved, uncharacterized* (C1orf177)		+/?	SPU_026146

**49**	98446	KOG3078	Nucleotide transport and metabolism	Adenylate kinase (adk)	Adenylate kinase-like protein		+/?	SPU_010767

**51**	181253	KOG1413	Carbohydrate transport and metabolism	*N*-acetylglucosaminyltransferase	*O*-linked-mannose beta-1,2-*N*-acetylglucosaminyltransferase		+/?	SPU_008219

**54**	163356	KOG0306	RNA processing and modification	WD40, coiled-coil	WD repeat domain 49	X	+/?	SPU_026011

**60**	237587	KOG0388	Replication, recombination and repair	Coiled-coil	Coiled-coil 74	X	**+/+**	SPU_018584

**62**	244599	KOG0531	Signal transduction mechanisms	LRR (Leucine-Rich Repeat)	Leucine rich repeat containing 48, phosphatase	X	+/?	SPU_006598

**63**	200285	KOG3577	Signal transduction mechanisms	Frizzled, C345C	sFRP1		+/+	SPU_011271

**68**	237703	KOG4193	Signal transduction mechanisms	GPCR proteolytic site, TM	Latrophilin		+/?	SPU_012362

**69**	235461	KOG1399	Secondary metabolites biosynthesis, transport and catabolism	Flavin-containing monooxygenase	Flavin-containing monooxygenase	X	+/?	SPU_007044

**71**	242796	*No KOG*	- - - - - - - -	*None*	Conserved, uncharacterized (C7orf31)		+/?	SPU_009640

**74**	30871	KOG0245	Cytoskeleton	Kinesin motor catalytic domain, FHA	Kinesin-like protein KIF16B	X	+/?	SPU_026237

**75**	86487	KOG4713	Signal transduction mechanisms	CDK2AP	CDK2-associated protein 1		+/?	SPU_004653

**77**	237378	KOG4115	Cell motility	RobLC7 domain	Dynein light chain roadblock-type 2	X	+/?	SPU_003137

**78**	237330	*No KOG*	- - - - - - - -	*None*	*Conserved, uncharacterized*		+/?	SPU_028435

**79**	59658	*No KOG*	- - - - - - - -	DUF4542	*Conserved, uncharacterized* (C17orf98)		+/?	SPU_004558

**80**	209931	*No KOG*	- - - - - - - -	*None*	*Conserved, uncharacterized* (C9orf135)	X	+/?	SPU_017778

**81**	239479	*No KOG*	- - - - - - - -	DUF3504	*Conserved, uncharacterized* (KIAA1958)		+/?	SPU_021568

**84**	205233	*No KOG*	- - - - - - - -	*None*	*Conserved, uncharacterized* (C9orf116)	X	+/?	SPU_004486

**85**	120202	KOG0667	General function prediction only	S/T protein kinase catalytic domain	Dual-specificity tyrosine-(*Y*)-phosphorylation regulated kinase DYRK4	X	+/?	SPU_012899

**90**	195162	*No KOG*	- - - - - - - -	Coiled-coil	Coiled-coil domain containing 105, tektin	X	+/?	SPU_002424

**92**	11327	*No KOG*	- - - - - - - -	*None*	*Conserved, uncharacterized* (FLJ43738)	X	**+/+**	SPU_005267

**95**	93809	KOG0161	Cytoskeleton	Coiled-coil	Coiled-coil 121	X	+/?	SPU_026895

**97**	183962	KOG3577	Signal transduction mechanisms	Frizzled, FRI (CRD)	Frizzled 5/8		+/+	SPU_022916

**101**	113661	KOG3961	Function unknown	ParcG	PARK2 co-regulated-like	X	+/?	SPU_012917

**103**	93943	KOG0161	Cytoskeleton	Coiled-coil, IQ motif (calmodulin binding)	IQ motif containing D	X	+/?	SPU_002424

**104**	218953	KOG1909	Signal transduction mechanisms	LRR	Ran GTPase-activating protein 1		+/?	SPU_004276

**110**	242938	*No KOG*	- - - - - - - -	TauD	Taurine catabolism dioxygenase TauD/TfdA		**−**	

**113**	234547	KOG4280	Cytoskeleton	Kinesin motor domain	Kinesin-like protein KIF9	X	+/?	SPU_000875

**114**	232308	KOG3079	Nucleotide transport and metabolism	*None*	Adenylate kinase 5 or 8	X	+/?	SPU_019553

**117**	223606	*No KOG*	- - - - - - - -	IQ motif, coiled-coil	Spermatogenesis-associated protein 17		+/?	SPU_023743

**124**	218233	KOG0274	General function prediction only	F-box, WD40	F-box and WD repeat domain containing 7	X	+/?	SPU_015976

**125**	164988	KOG4682	General function prediction only	*None*	BTB (POZ) domain containing 16		+/?	SPU_015356

**130**	171968	KOG3882	General function prediction only	Tetraspanin	Tetraspanin (33)	X	+/?	SPU_027747

**132**	83869	*No KOG*	- - - - - - - -	Glycoside hydroxylase	Endoglucanase		+/?	SPU_021602

**137**	182272	KOG3961	Function unknown	ParcG	PARK2 co-regulated	X	**+/+**	SPU_004619

**139**	160170	*No KOG*	- - - - - - - -	Coiled-coil, THEG	Testicular haploid expressed gene protein-like		**+/+**	SPU_026963

**142**	162410	*No KOG*	- - - - - - - -	DUF3695	*Conserved, uncharacterized* (C1orf194)	X	**+/+**	SPU_013076

**145**	199721	KOG3645	Signal transduction mechanisms	Neurotransmitter-gated ion channel ligand binding domain, neurotransmitter-gated ion channel transmembrane domain (Pfam)	Nicotinic Acetylcholine Receptor alpha		**+/+**	SPU_001774

**146**	131533	KOG3714	Posttranslational modification, protein turnover, chaperones	Zn dependent metalloprotease, MAM	Meprin	X	**+/+**	SPU_004114

**147**	79471	KOG3539	Extracellular structures	Spondin, TSP1	Spondin-1	X	+/?	SPU_009594

**148**	123439	*No KOG*	- - - - - - - -	Scavenger Receptor Cysteine-rich	Galectin 3 binding protein	X	−	

**149**	243308	*No KOG*	- - - - - - - -	Tubulin tyrosine ligase	Tubulin tyrosine ligase-like 9	X	+/?	SPU_000277

**151**	81127	KOG3676	Inorganic ion transport and metabolism	Ankyrin, PKD channel	TRPV channel	X	**+/+**	SPU_007504

**153**	240906	*No KOG*	- - - - - - - -	Coiled-coil	Stathmin 4, Nucleolar Protein 9		+/?	SPU_008203

**154**	81173	*No KOG*	- - - - - - - -	*None*	*Conserved, uncharacterized*		**+/+**	SPU_005437

**155**	168814	KOG4364	Chromatin structure and dynamics	Coiled-coil	Coiled-coil domain containing 81	X	+/?	SPU_021664

**156**	90973	KOG1496	Energy production and conversion	Coiled-coil, lactate/malate dyhdrogenase NAD binding and C-terminal domains	Malate dehydrogenase	X	**+/+**	SPU_015928

**159**	117995	KOG0032	Signal transduction mechanisms	S/T kinase catalytic domain	DAP kinase-related apoptosis-inducing protein kinase 1	X	+/?	SPU_028649

**161**	240545	*No KOG*	- - - - - - - -	KIAA1430	*Conserved, uncharacterized* (C17orf105)	X	+/?	SPU_010239

**162**	245773	KOG1375	Cytoskeleton	Tubulin GTPase, Tubulin C-terminal	Beta tubulin	X	**+/+**	SPU_000062

**165**	238199	KOG4415	Function unknown	Coiled-coil	*Conserved, uncharacterized*		+/?	SPU_011316

**166**	245865	KOG3508	General function prediction only	PKC conserved region 2, RasGAP, DUF 3498	Disabled homolog 2-interacting protein	X	−	

**167**	34056	KOG0819	Intracellular trafficking, secretion, and vesicular transport	Annexin	Annexin A	X	**+/+**	SPU_019139

**168**	41471	KOG0200	Signal transduction mechanisms	Tyrosine kinase	FGF receptor	X	−	

**169**	208307	KOG0531	Signal transduction mechanisms	LRR	Protein phosphatase 1 regulatory subunit 7	X	+/?	SPU_012637

**171**	245626	KOG2450	Energy production and conversion	Aldehyde dyhydrogenase	ALDH, ALDH1b	X	+/−	SPU_007284

**175**	143747	KOG3940	Function unknown	C2HC Zn finger	Zn finger C2HC domain containing protein 1 C	X	+/?	SPU_007461

**180**	3074	KOG4308	Function unknown	LRR	*Conserved, uncharacterized* (C14orf166B); *potentially part of ao46*	X	+/?	SPU_005282

**181**	245069	*No KOG*	- - - - - - - -	NADH dehydrogenase, FAD-containing subunit	*Conserved, uncharacterized* (C20orf26)	X	+/?	SPU_018537

**182**	208725	*No KOG*	- - - - - - - -	DUF4562	*Conserved, uncharacterized* (C4orf45)		−	

**189**	94003	KOG0490	General function prediction only	HOX	NVHD146-paired class homeobox protein OR Q50-6 [*Nematostella vectensis*]	X	−	

**190**	212596	KOG3885	Signal transduction mechanisms	FGF	Fibroblast growth factor 1E [*Nematostella vectensis*]		**+/+**	SPU_006242

**193**	165815	KOG3585	Transcription	T-Box	Tbx4/5 protein [*Podocoryne carnea*]	X	**+/+**	SPU_023386

**194**	65438	KOG2294	Transcription	Forkhead	Forkhead box J1b [*Danio rerio*]	X	**+/+**	SPU_027969

**195**	153628	KOG0490	General function prediction only	HOX	K50-5 [*Nematostella vectensis*]; DMBXf-paired class homeobox protein [*Nematostella vectensis*],	X	−	

## Appendix A Supplementary materials

Supplementary data: **Table S1: A subset of previously identified apical organ genes is recovered in the microarray** Note that in Marlow et al., 2009, the sequence annotated as *NvNCAM2* is producing the expression pattern that is described as *NvNCAM3* (and vice versa).
