# Supplementary material for: Molecular characterization of the apical organ of the anthozoan Nematostella vectensis
Source: Dev Biol. 2015 Feb 1;398(1):120–33. doi: 10.1016/j.ydbio.2014.11.019 (PMC4300403; doi:10.1016/j.ydbio.2014.11.019)
Supplement: Supplementary file 1 — Supplementary data: Table S1: A subset of previously identified apical organ genes is recovered in the microarray Note that in Marlow et al., 2009, the sequence annotated as NvNCAM2 is producing the expression pattern that is described as NvNCAM3 (and vice versa). [file mmc1.doc]

|  | known *Nematostella* AO genes | identified in microarray? | additional expression domains? | reference |
| --- | --- | --- | --- | --- |
|  | *hoxF/Anthox1* | yes | yes | Finnerty et al., 2004 |
|  | *FGFa2* | yes | no | Rentzsch et al., 2008 |
|  | *lhx6/8* | yes | no | Srivastava et al., 2010 |
|  | *NCAM2* | yes | yes | Marlow et al., 2009 |
|  | *noggin* | yes | yes | Matus et al., 2006 |
|  | *fz5/8* | yes | no | Kumburegama et al., 2011 |
|  | *gremlin* | yes | yes | Rentzsch et al., 2006 |
|  | *COE* | no | no | Pang et al., 2004 |
|  | *FGFa1* | no | no | Matus et al., 2007 |
|  | *soxB1* | no | yes | Magie et al., 2005 |
|  | *FGFRa* | no | yes | Matus et al., 2007 |
|  | *netrin* | no | yes | Matus et al., 2006 |
|  | *irx* | no | no | Sinigaglia et al., 2013 |
|  | *nanos2* | no | yes | Extavour et al., 2005 |
|  | *nk3* | no | no | Marlow et al., 2013 |

**Refernce**

Marlow, H.Q., Srivastava, M., Matus, D.Q., Rokhsar, D., Martindale, M.Q. 2009. Anatomy
and development of the nervous system of *Nematostella vectensis*, an anthozoan cnidarian.
Dev Neurobiol. 69(4):235-54.
